# 629. Evaluation of Neurocysticercosis Healthcare Provider Diagnostic Practices in Houston, Texas

**DOI:** 10.1093/ofid/ofae631.194

**Published:** 2025-01-29

**Authors:** Theresa Sepulveda, Fernando H Centeno, Jose Serpa, Jill E Weatherhead, Eva Clark

**Affiliations:** Baylor College of Medicine, Houston, TX; Baylor College of Medicine, Houston, TX; Baylor College of Medicine, Houston, TX; Baylor College of Medicine, Houston, TX; Baylor College of Medicine, Houston, TX

## Abstract

**Background:**

Neurocysticercosis (NCC) is a neglected tropical disease caused by infection with the pork tapeworm *Taenia solium* that affects 2-8 million people globally and is responsible for ∼30% of epilepsy cases in endemic countries. Based on case series data, > 4,000 new NCC cases are estimated to occur annually in the US. The gold standard for NCC diagnosis as outlined in the Infectious Disease Society of America (IDSA) guidelines is neuroimaging with computed tomography (CT: to detect calcifications) and magnetic resonance imaging (MRI: to detect viable disease), followed by confirmatory serologic testing with the enzyme-linked immunoelectrotransfer blot (EITB) or other reliable assay. Our study aims to describe healthcare provider diagnostic practices at a public healthcare system serving at-risk patients in Texas.

**Figure:**
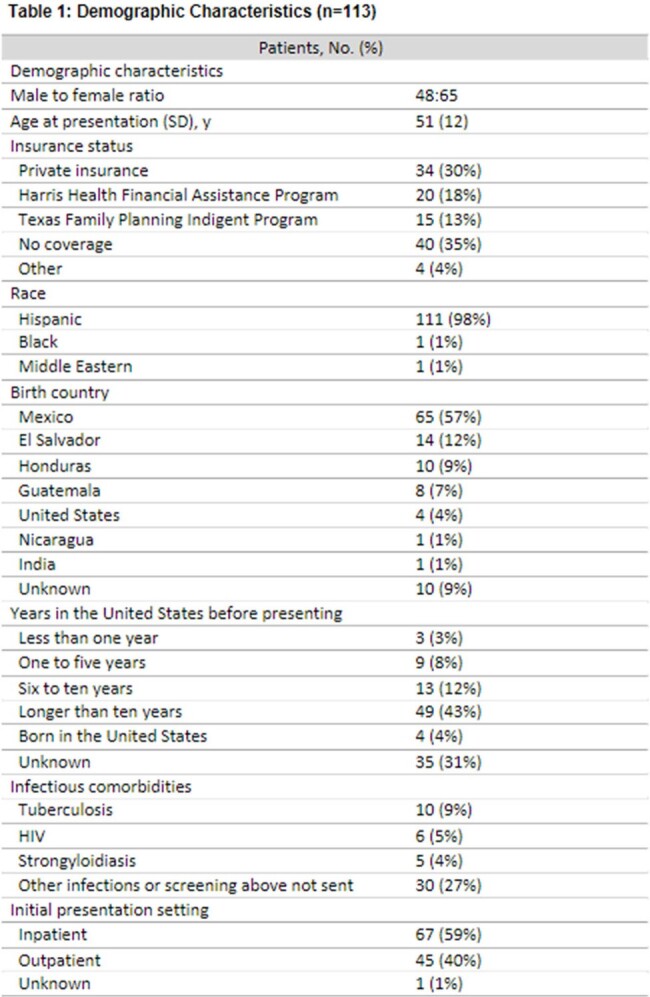

**Methods:**

We retrospectively identified patients ≥ 18 years old tested for NCC in the Harris Health System [HHS] between 2017-2021, systematically extracted variables from the patients’ electronic medical records, and recorded this data via a REDCap instrument. We classified NCC patients by NCC type and performed descriptive statistics.

**Results:**

We identified 113 patients with NCC (Table 1). Family medicine (n=44, 39%) or internal medicine (n=29, 26%) providers evaluated most patients in their first diagnostic encounter. Presenting signs/symptoms included seizure (n=75, 66%) and chronic headaches (n=77, 68%). 77% of patients with calcified disease and 88% with viable disease were diagnosed within 5 years after symptom onset. Only 63% of patients had both CT and MRI completed. Zero initial providers ordered EITB confirmatory serologic testing; n=4, 3% ordered other types of serologic testing. Only five patients ultimately had EITB sent; of those, two tested positive. While 47% of initial providers referred patients to neurology and 18% referred to neurosurgery, only 35% referred to tropical medicine or infectious diseases specialties.

**Conclusion:**

Improved education of frontline healthcare providers on NCC presentation and diagnosis may reduce time to NCC diagnosis. Future work should target the need for structured education and standardization of diagnostic practices especially amongst frontline healthcare providers serving at-risk communities in the US.

**Disclosures:**

**All Authors**: No reported disclosures

